# Involvement of HMGB1-mediated ferroptosis in systemic diseases

**DOI:** 10.3389/fcell.2025.1676941

**Published:** 2025-10-03

**Authors:** Lijie Lv, Yue Wang, Xuan Lv, Qiuli Miao

**Affiliations:** ^1^ Department of Rehabilitation, Lequn Branch, The First Hospital of Jilin University, Changchun, China; ^2^ Department of Nursing, Affiliated Hospital of Changchun University of Traditional Chinese Medicine, Changchun, China; ^3^ Department of Anorectal Diseases, Affiliated Hospital of Changchun University of Traditional Chinese Medicine, Changchun, China; ^4^ Department of Pharmacy, Lequn Branch, The First Hospital of Jilin University, Changchun, China

**Keywords:** ferroptosis, HMGB1, systemic diseases, regulated cell death, lipid peroxidation, oxidative stress, inflammation

## Abstract

Ferroptosis is a distinct, iron-dependent form of regulated cell death characterized by lipid peroxidation and redox imbalance. High-mobility group box 1 (HMGB1), a nuclear protein with strong immunomodulatory capacity, has emerged as a key regulator in ferroptosis-related pathologies. Acting both as a downstream effector released during ferroptotic cell death and as an upstream amplifier of inflammation, immune activation, and metabolic dysfunction, HMGB1 plays a context-dependent dual role in disease progression. In tumor settings, HMGB1-mediated ferroptosis enhances antitumor immunity and suppresses tumor growth. Conversely, in non-neoplastic diseases, such as ischemia-reperfusion injury and inflammatory disorders, HMGB1 release exacerbates tissue damage and immune dysregulation. This review comprehensively summarizes the molecular mechanisms of HMGB1-induced ferroptosis, including its regulation via autophagy–ferritinophagy and redox signaling pathways. We further examine how the HMGB1–ferroptosis axis contributes to systemic diseases affecting the respiratory, digestive, nervous, circulatory, urinary, locomotor, endocrine, reproductive, and immune systems. Finally, we discuss emerging therapeutic strategies that target this axis with an emphasis on disease-specific interventions modulating ferroptosis, inflammation, and immune responses.

## 1 Introduction

Ferroptosis is a unique, iron-dependent form of regulated cell death, first described in 2012, which is biochemically, genetically, and morphologically distinct from apoptosis, necrosis, and autophagy. It is characterized by the accumulation of intracellular iron and excessive lipid peroxidation (LPO) (Dixon et al., 2012). Unlike traditional forms of cell death, ferroptosis lacks classical morphological features characteristic of apoptosis or necrosis, such as chromatin condensation or cell swelling, and instead exhibits a relatively unchanged nucleus size, reduced cell volume, increased membrane density, loss of mitochondrial cristae, and occasional mitochondrial rupture (Yang et al., 2025). Furthermore, transmission electron microscopy studies have identified electron-lucent cytoplasm and an electron-lucent nucleus as morphological hallmarks of ferroptosis, with the latter clearly distinguishing it from necroptosis (Miyake et al., 2020). Biochemically, ferroptosis is driven by impaired antioxidant defenses, particularly the depletion of glutathione (GSH) and the consequent inactivation of glutathione peroxidase 4 (GPX4), which leads to the unchecked accumulation of lipid reactive oxygen species (ROS). Simultaneously, excessive free iron may promote the formation of ROS through the Fenton reaction. If these oxidative byproducts are not efficiently eliminated, they damage intracellular proteins, lipids, and nucleic acids, ultimately leading to cell death (Yang et al., 2025; Wang et al., 2024a). Recent studies have implicated ferroptosis in a wide range of diseases, including respiratory diseases (Bao et al., 2022), neurodegenerative diseases (Liu et al., 2023), ischemia-reperfusion injury (IRI) (Stamenkovic et al., 2021), and cancer (Dai et al., 2020).

High-mobility group box 1 (HMGB1) is a highly conserved non-histone nuclear protein composed of two DNA-binding domains and an acidic C-terminal tail (Chen R. et al., 2022). Depending on its subcellular localization, HMGB1 performs diverse functions. In the nucleus, HMGB1 acts as a DNA chaperone, participating in DNA replication, repairing, transcription, and maintaining genome stability; in the cytoplasm, it promotes autophagy (Chen R. et al., 2022; Wen et al., 2019). Under cellular stress, HMGB1 translocates to the plasma membrane or extracellular space, where it acts as a prototypical damage-associated molecular pattern (DAMP), activating immune responses through interaction with receptors such as the receptor for advanced glycation end products (RAGE, also known as AGER) and Toll-like receptors (TLRs) 2 and 4 (Shao et al., 2023; Allegra et al., 2023). HMGB1 can be actively secreted by immune cells such as macrophages and dendritic cells (DCs) or passively released by necrotic and dying cells. Its activity and release are tightly regulated by post-translational modifications (e.g., acetylation, phosphorylation, methylation, and ADP-ribosylation) and ROS/redox signaling (Wen et al., 2019; Shao et al., 2023). Extracellular HMGB1 not only promotes inflammation but also modulates metabolic responses and contributes to various pathological processes, including cancer, infection, tissue damage, and cell death pathways such as apoptosis, necrosis, necroptosis, pyroptosis, ferroptosis, alkaliptosis, and cuproptosis (Chen R. et al., 2023).

Recently, the role of HMGB1 and its regulatory mechanisms in ferroptosis have attracted increasing attention. Experimental studies have shown that ferroptosis inducers promote HMGB1 release and that HMGB1 translocates from the nucleus to the cytosol and ultimately to the extracellular space via compromised nuclear membrane integrity in ferroptotic cells. This suggests that nuclear membrane damage precedes cytoplasmic membrane rupture during ferroptosis (Miyake et al., 2020). Additionally, HMGB1 inhibition or RAGE deficiency attenuates the ferroptosis-induced inflammatory response in macrophages, indicating that targeting HMGB1 release may limit iron-driven inflammatory responses during ferroptosis (Wen et al., 2019). Notably, abnormal HMGB1 functions as a “double-edged sword,” playing both pro-tumor and anti-tumor roles depending on the cancer type and stage, and acting as a facilitator of ferroptotic cancer cell death while also promoting pathological inflammation in IRI and other diseases (Miyake et al., 2020; Shao et al., 2023; Chen R. et al., 2023).

In this review, we comprehensively explore the dual role of HMGB1-induced ferroptosis across systemic diseases, including diseases of the respiratory, digestive, nervous, circulatory, urinary, locomotor, endocrine, reproductive, and immune systems. We also discuss the underlying molecular mechanisms and evaluate emerging therapeutic strategies targeting the HMGB1–ferroptosis axis.

## 2 Molecular mechanisms of HMGB1-Induced ferroptosis

### 2.1 Canonical pathways and autophagy-mediated regulation of ferroptosis

Ferroptosis is a form of regulated cell death characterized by the iron-dependent accumulation of lipid peroxides resulting from the disruption of antioxidant defenses, oxidative stress, and dysregulated iron metabolism (Dixon et al., 2012). Environmental insults such as hypoxia, ischemia, and pharmacological agents that impair cellular redox systems are well-established triggers of ferroptosis (Hu et al., 2021). A hallmark biochemical feature of ferroptosis is the Fenton reaction, in which ferrous ions (Fe^2+^) catalyze the formation of hydroxyl radicals that attack polyunsaturated fatty acid (PUFA)-containing phospholipids in cellular membranes, promoting widespread LPO and membrane damage. However, recent studies suggest that the Fenton reaction alone may be insufficient to fully amplify ferroptotic signaling, as the endogenous levels of PUFA-containing lipids may not be adequate to reach the threshold for lethal LPO. Zhang et al. proposed that a positive feedback loop involving LPO, protein kinase C βII (PKCβII), and acyl-CoA synthetase long-chain family member 4 (ACSL4) enhances the biosynthesis of PUFA-containing phospholipids. This PKCβII–ACSL4 axis cooperates with the Fenton reaction to reinforce LPO, underscoring that both pathways are essential for the execution of ferroptosis (Zhang et al., 2022). In parallel, the canonical ferroptosis pathway centers on the system X_c_^−^–GSH–GPX4 axis (Dixon et al., 2012; Zhao et al., 2023). System X_c_^−^, a cystine/glutamate antiporter composed of solute carrier family 7 member 11 (SLC7A11/xCT) and solute carrier family 3 member 2 (SLC3A2), mediates the import of extracellular cystine, which is subsequently reduced to cysteine for GSH synthesis (Hu et al., 2021). GSH serves as a critical cofactor for GPX4, an essential phospholipid peroxidase that detoxifies lipid hydroperoxides and prevents their accumulation. Inhibition of either system X_c_^−^ or GPX4 disrupts cellular redox homeostasis, leading to ROS accumulation and unchecked LPO, ultimately triggering ferroptotic cell death. Erastin and RSL3 are specific inhibitors of system Xc^−^ and GPX4, respectively, and are the most used classic reagents to induce ferroptosis (Dixon et al., 2012; Miyake et al., 2020; Zhao et al., 2023).

Although initially described as autophagy-independent (Dixon et al., 2012), increasing evidence suggests that autophagy plays a critical, context-dependent role in modulating ferroptotic sensitivity. Autophagy is an evolutionarily conserved lysosomal degradation pathway that facilitates the removal of damaged organelles and proteins. While moderate autophagy promotes cell survival by recycling essential biomolecules, excessive or selective autophagy may enhance ferroptosis by degrading protective components and disrupting intracellular iron homeostasis. Selective forms of autophagy such as ferritinophagy, lipophagy, clockophagy, and chaperone-mediated autophagy have been implicated in ferroptosis progression. These processes selectively degrade critical substrates, including ferritin, lipid droplets, circadian clock regulators (e.g., aryl hydrocarbon receptor nuclear translocator like [ARNTL]), and GPX4, thereby increasing labile iron pools, enhancing LPO, reducing antioxidant capacity, and ultimately compromising membrane integrity (Hu et al., 2021; Chen et al., 2021a; Chen X. et al., 2023).

Ferritinophagy is one of the most studied pathways linking autophagy to ferroptosis. It is mediated by nuclear receptor coactivator 4 (NCOA4), which selectively binds to ferritin heavy chain 1 (FTH1) and delivers ferritin to the autophagosome for lysosomal degradation. This process releases free Fe^2+^, fuelling the Fenton reaction and accelerating ferroptotic signaling (Wang et al., 2024b). Poly (rC)-binding protein 2 (PCBP2), an iron chaperone and RNA-binding protein, restricts ferroptosis by facilitating iron storage via ferritin loading and stabilizing system Xc^−^ function through SLC7A11 mRNA protection. Downregulation of PCBP2 impairs these functions, promoting iron-dependent oxidative damage and ferroptotic vulnerability (Jia et al., 2025). In parallel, autophagy also regulates transferrin receptor 1 (TfR1/TFRC), a surface glycoprotein that facilitates cellular iron uptake via transferrin binding. Its autophagic modulation is context-dependent: degradation of TfR1 suppresses ferroptosis, while its upregulation promotes it. The mammalian target of rapamycin (mTOR)/tristetraprolin (TTP) pathway has been shown to inhibit TfR1 mRNA translation under low-iron conditions as a pro-survival adaptation (Wei et al., 2024). Furthermore, lysosomal cell death mediated by signal transducer and activator of transcription 3 (STAT3) contributes to ferroptosis through increased cathepsin expression and activity (Wen et al., 2019). All these pathways promote iron overload, ROS generation, and ultimately ferroptosis.

Beyond the system X_c_^−^–GSH–GPX4 axis, lipophagy mediated by RAB7A promotes neutral lipid utilization and contributes to LPO. Autophagy-related proteins such as Beclin-1 (BECN1) and p62 are also involved. BECN1 plays a central role in autophagosome formation and maturation and can bind SLC7A11 to inhibit system X_c_^−^, causing GSH depletion and ferroptosis (Wen et al., 2019). p62 mediates selective autophagy by directing damaged components to autophagosomes. In ferroptotic cells, BECN1 is upregulated and p62 is downregulated, confirming autophagy activation (Gechang et al., 2024).

Taken together, ferroptosis can be initiated by both autophagy-dependent and -independent pathways. It is orchestrated by a finely balanced network of lipid oxidation, iron metabolism, and antioxidant defenses. While the system X_c_^−^–GSH–GPX4 axis forms the core pathway, autophagy, particularly its selective forms, amplifies ferroptosis by modulating intracellular redox status and iron availability. In various pathological conditions including cancer, liver fibrosis, IRI, and acute kidney injury, autophagy-dependent ferroptosis has emerged as a key contributor to disease progression (Wei et al., 2024).

### 2.2 Autophagy-dependent HMGB1 translocation and proinflammatory signaling in ferroptosis

As research on ferroptosis progresses, HMGB1 has emerged as a pivotal regulator linking oxidative cell death, iron homeostasis, and inflammation. Under physiological conditions, HMGB1 resides predominantly in the nucleus, where it binds chromatin to regulate DNA replication, repair, and transcription. In response to ferroptotic stress, however, HMGB1 translocates to the cytoplasm and is subsequently released extracellularly. Miyake et al. demonstrated that lipid hydroperoxide-induced disruption of the nuclear membrane before plasma membrane rupture during ferroptosis, enabling early passive HMGB1 release into the cytoplasm (Miyake et al., 2020). Its intracellular translocation is regulated by ROS and the RAS (Rat sarcoma viral oncogene homolog)–JNK (c-Jun N-terminal kinase)/p38 mitogen-activated protein kinase (MAPK) signaling cascade (Chen et al., 2021b; Kepp et al., 2023). Additional findings in methotrexate (MTX)-induced liver injury revealed that this ROS-dependent translocation facilitates autophagic flux by dissociating the BECN1/B cell lymphoma 2 (Bcl-2) complex, thereby promoting autophagy, ferritinophagy, and ferroptotic injury (Wang et al., 2024b).

In addition to passive translocation, HMGB1 can be actively secreted through tightly regulated mechanisms. This process begins with HMGB1 acetylation, which disrupts chromatin tethering and permits nuclear export (Shokr and Eladawy, 2025). Histone deacetylase (HDAC) inhibition promotes this acetylation, enhancing HMGB1 nuclear-cytoplasmic transport (Wen et al., 2019). Moreover, suppression of PCBP2 increases the transcriptional activity of hypoxia-inducible factor 1α (HIF1α) and its coactivator E1A binding protein p300 (p300), further facilitating HMGB1 acetylation and secretion (Jia et al., 2025). Once in the cytoplasm, autophagy-related proteins ATG5 and ATG7 mediate HMGB1 incorporation into autophagosomes or lysosomes, which then fuse with the plasma membrane to enable its extracellular release (Wen et al., 2019; Shokr and Eladawy, 2025). This autophagy-dependent, active secretion occurs before cell membrane rupture and is thus distinct from the passive leakage observed during the terminal stages of ferroptosis when excessive LPO compromises membrane integrity (Miyake et al., 2020; Shokr and Eladawy, 2025). Notably, the nuclear factor erythroid 2–related factor 2 (Nrf2)-pirin (PIR) pathway acts as a negative regulator: Nrf2-induced PIR expression limits DNA damage and suppresses HMGB1-mediated autophagy, thereby restraining HMGB1 release and subsequent ferroptosis (Hu et al., 2021).

Once released, HMGB1 functions as a prototypical DAMP, binding to pattern recognition receptors such as RAGE and Toll-like receptors (TLRs). These interactions activate inflammatory signaling cascades, including nuclear factor kappa B (NF-κB), thereby establishing a self-amplifying ferroptotic-inflammatory circuit (Li et al., 2025). While RAGE, but not TLR4, is essential for HMGB1-induced tumor necrosis factor-α (TNF-α) secretion in macrophages exposed to ferroptotic cells (Wen et al., 2019), TLR4–STAT3 signaling is activated in M1 macrophages by HMGB1 released from ferroptotic M2 macrophages, promoting inflammation and disrupting M1/M2 polarization (Feng et al., 2024). HMGB1 also contributes to ferroptosis sensitivity through the TLR4–Yes-associated protein (YAP) axis, as observed in neutrophil extracellular trap (NET)-associated responses (Zhao P. et al., 2024). Phosphorylated p38 MAPK promotes the nuclear translocation of YAP, which transcriptionally upregulates *TFRC* and *ALOXE3*, enhancing ferroptosis through iron accumulation and LPO (Wu et al., 2023). Qi et al. further identified a positive feedback loop in which extracellular HMGB1 promotes TfR1-mediated iron uptake in neighboring cells, facilitating transcellular ferroptotic propagation and tissue injury (Wei et al., 2024). The Nrf2–Heme oxygenase-1 (HO-1) pathway modulates HMGB1 activity as well. HO-1, an antioxidant enzyme regulated by Nrf2, catalyzes the degradation of heme into carbon monoxide, biliverdin, and ferrous iron (Shokr and Eladawy, 2025). Collectively, inhibition of HMGB1 reduces Fe^2+^ levels, enhances antioxidant defense via GSH/GPX4 and Nrf2/HO-1 signaling, and mitigates ferroptotic injury (Wang et al., 2019).

Importantly, the immunological consequences of HMGB1 release during ferroptosis are profoundly influenced by its timing. Wiernicki et al. delineated ferroptosis into three stages: initial (LPO), intermediate (ATP release), and terminal (HMGB1 release), and discovered that only early-stage ferroptotic cells effectively promoted DC maturation. In contrast, late-stage ferroptotic cells with passive HMGB1 release exhibited reduced phagocytosis and antigen cross-presentation by DCs, ultimately failing to induce protective antitumor immunity. These findings suggest that while HMGB1 acts as a key DAMP in ferroptosis, the stage-specific dynamics of its release may differentially modulate immune responses, with early-phase modulation favoring immunogenicity and late-phase leakage potentially dampening adaptive immunity (Wiernicki et al., 2022).

The HMGB1–ferroptosis axis exhibits context-dependent duality. For example, in lipopolysaccharide (LPS)-induced acute kidney injury, downregulation of HMGB1 and NCOA4 expression inhibits ferritinophagy, thereby alleviating ferroptosis in renal tubular cells (Tang et al., 2021). This suggests anti-inflammatory effects of inhibiting HMGB1 signaling in non-malignant tissues. In contrast, in ovarian cancer (OC), decreased NCOA4-mediated ferritinophagy suppresses ROS accumulation and prevents mitochondrial damage and HMGB1 release, which facilitates immune evasion and tumor progression (Jin et al., 2022). Thus, inhibition of ferroptosis and HMGB1 release in cancer cells may inadvertently promote tumor development.

**FIGURE 1:**
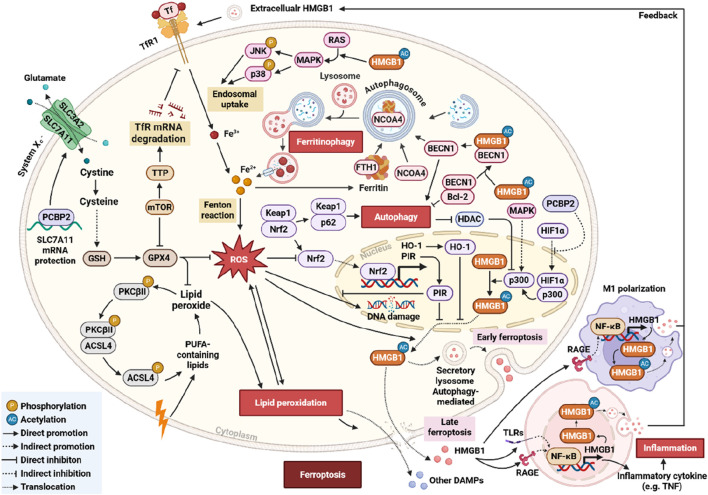
Schematic representation of the HMGB1–ferroptosis regulatory network and its autophagy-mediated modulation. This diagram shows HMGB1 regulating ferroptosis via autophagy-dependent/-independent pathways, involving ROS-induced LPO, the system Xc^−^–GSH–GPX4 pathway, NCOA4-mediated ferritinophagy, BECN1 and p62 in autophagy, HMGB1 translocation and secretion, extracellular HMGB1 activating NF-κB signaling, and negative regulation by the Nrf2–HO-1–PIR pathway.

In summary, HMGB1 governs ferroptosis through stage-specific release mechanisms and its engagement with autophagy–ferritinophagy signaling. Acting as both an intracellular stress sensor and an extracellular immune modulator, HMGB1 connects redox imbalance, iron metabolism, and inflammation (Figure 1). Its divergent effects—promoting immune surveillance in tumors *versus* aggravating inflammatory injury in non-malignant tissues—highlight its dual role as both a biomarker and a therapeutic target in ferroptosis-related diseases.

## 3 Ferroptosis–HMGB1 axis in systemic diseases

### 3.1 Respiratory system diseases

#### 3.1.1 Acute lung injury (ALI) and lung ischemia-reperfusion injury (IRI)

Acute lung injury (ALI) is a severe condition highly susceptible to ferroptosis due to its high metabolic demand and oxygen exposure. During ferroptosis, HMGB1 translocates and promotes oxidative damage and inflammation (Abdallah et al., 2025). Silencing HMGB1 reduces ferroptosis markers, such as ROS, malondialdehyde (MDA), and Fe^2+^, while restoring SLC7A11/GPX4 levels and activating the Nrf2–HO-1 pathway. These effects are reversed by the Nrf2 inhibitor ML385 (Abdallah et al., 2025; Jia et al., 2024). Ferrostatin-1 (Fer-1), a ferroptosis inhibitor, further confirms this link. Additionally, HMGB1 regulates heat shock protein beta-1 (HSPB1), which stabilizes mitochondrial function and limits iron uptake. Loss of HSPB1 exacerbates ferroptosis via interleukin-6 (IL-6) overexpression and Kelch-like ECH-associated protein 1 (Keap1)-Nrf2 axis disruption (Abdallah et al., 2025). Lung ischemia-reperfusion injury (IRI) is the primary cause of graft dysfunction after transplantation yet lacks effective therapies. HMGB1-mediated ferroptosis in pulmonary endothelial cells exacerbates sterile inflammation during IRI (Chongwu Li et al., 2024). In rodent models, IRI upregulates arachidonate ALOX12 and its metabolite 12-hydroxyeicosatetraenoic acid (12-HETE), inducing endothelial ferroptosis and HMGB1 release. This subsequently activates the TLR4/myeloid differentiation primary response 88 (MYD88) pathway in neutrophils, triggering the formation of neutrophil extracellular traps (NETs) and worsening inflammation. ALOX12 knockout or inhibition (e.g., ML355) significantly attenuated ferroptosis, NET formation, and lung tissue injury (Chongwu Li et al., 2024). Additionally, the lipid mediator lipoxin A4 (LxA4) protects against IRI via formyl peptide receptor 2 (FPR2) by restoring GPX4/Nrf2, reducing LPO and proinflammatory cytokines including HMGB1-an effect absent in FPR2-deficient mice (Sharma et al., 2022). These findings implicate that the ALOX12/12-HETE-ferroptosis-HMGB1-NET axis represents a key pathogenic mechanism and therapeutic target.

#### 3.1.2 Asthma

Asthma, a chronic inflammatory airway disease, encompasses eosinophilic (T2-high) and neutrophilic (T2-low) subtypes, with ferroptosis and HMGB1 driving severe steroid-resistant phenotypes (Allegra et al., 2023). In eosinophilic asthma, type 2 cytokines IL-13 suppresses GPX4 and induces 15-lipoxygenase (ALOX15), promoting LPO and airway remodeling via phospholipase B1 (PEBP1)/rapidly accelerated fibrosarcoma (RAF)/extracellular signal-regulated kinase (ERK) signaling. HMGB1 released from damaged epithelium activates RAGE/TLR4, amplifying eosinophilic inflammation (Allegra et al., 2023; Li Y. et al., 2023). Environmental toxins, such as dibutyl phthalate (DBP), exacerbate this pathway by inducing Fe^2+^ accumulation, LPO, and HMGB1/IL-33/ALOX15 upregulation-effects reversed by Fer-1 in murine models (Li Y. et al., 2023). In neutrophilic asthma, LPS/IL-13-induced ferroptosis triggers epithelial injury and neutrophil infiltration. Liproxstatin-1 (Lip-1) inhibits this process by restoring GPX4/SLC7A11, reducing ROS/MDA, and suppressing HMGB1/IL-33 release, thereby attenuating inflammation (Bao et al., 2022). Critically, the HMGB1-IL-33 axis bridges ferroptosis to immune dysregulation. ATP/ADP-driven activation of the P2Y purinoceptor 13 (P2Y13) receptor has been shown to promote the release of HMGB1 and IL-33, which fuels a feedback loop of inflammation and tissue injury (Allegra et al., 2023).

#### 3.1.3 Pulmonary hypertension (PH)

Pulmonary hypertension (PH) features pulmonary vascular remodeling, right ventricular hypertrophy, and elevated pulmonary arterial pressure. Ferroptosis in pulmonary artery endothelial cells (PAECs) and HMGB1-mediated inflammation contribute significantly to PH (Liao et al., 2023; Xie et al., 2022). In monocrotaline-induced PH models, PAECs exhibit LPO accumulation, GPX4/FTH1 downregulation, and NADPH oxidase 4 (NOX4) upregulation, resulting in HMGB1 release. This, in turn, activates TLR4/NLR family pyrin domain containing 3 (NLRP3) inflammasomes in macrophages, promoting IL-1β and IL-18 secretion and exacerbating vascular injury (Liao et al., 2023; Xie et al., 2022). Fer-1 alleviates vascular remodeling and inflammation by targeting this ferroptosis-HMGB1 axis (Xie et al., 2022). Additionally, peroxiredoxin 6 (PRDX6), a redox-regulating enzyme in PAECs, exerts protective effects by inhibiting ferroptosis, restoring GPX4/FTH1, and reducing HMGB1 release, thereby preserving endothelial integrity (Liao et al., 2023).

#### 3.1.4 Non-Small Cell Lung Cancer (NSCLC) and lung adenocarcinoma tumor (LUAD)

Non-Small Cell Lung Cancer (NSCLC) accounts for approximately 85% of lung cancer cases and remains highly lethal due to therapy resistance. The ferroptosis-HMGB1 axis plays a crucial role in NSCLC progression and antitumor immunity (Zhang et al., 2025; Boullosa et al., 2021). Zhang et al. demonstrated that resveratrol (RSV) inhibits NSCLC growth by stimulating the release of HMGB1-enriched extracellular vesicles (EVs), which induce platelet ferroptosis via ROS accumulation and impaired cystine transport (Zhang et al., 2025). Similarly, iridium (III) complexes trigger ferroptosis and autophagy in A549 cells through GSH depletion, MDA/LPO elevation, GPX4/ferritin downregulation, and BECN1-mediated ferritinophagy-all in an HMGB1-dependent manner (Gechang et al., 2024). In mutant p53 NSCLC, Boullosa et al. validated auranofin (AF) as a ferroptosis inducer, showing GPX4 inhibition, LPO induction, and Nrf2 resistance override. AF also promoted immunogenic cell death (ICD) with calreticulin (CRT) exposure, ATP/HMGB1 release, DC maturation, and immunosuppressive cytokine suppression (e.g., IL-6, TNF-α, TGF-β) (Boullosa et al., 2021). These studies highlight HMGB1 as a key mediator linking ferroptosis to ICD and immune activation, providing a promising strategy to overcome therapy resistance in NSCLC (Gechang et al., 2024; Boullosa et al., 2021).

Lung adenocarcinoma (LUAD), the most prevalent NSCLC subtype, often develops resistance to epidermal growth factor receptor tyrosine kinase inhibitor (EGFR-TKI) resistance and exhibits low immune checkpoint expression. Ferroptosis-related genes influence tumorigenesis, prognosis, and immunotherapy response by modulating the tumor immune microenvironment and mutation burden. Transcriptomic profiling identified ferroptosis subtypes (FS1-FS3), with FS1 linked to favorable prognosis and high immune infiltration, while FS3 is associated with increased HMGB1 expression and poor outcomes. Moreover, expression of ferroptosis-related tumor antigens (e.g., NOX4, PANX1, MTDH) correlates with enhanced antigen-presenting cell infiltration and ICD markers. These insights support the potential of ferroptosis-based subtyping and immunogenic markers for developing personalized mRNA cancer vaccines in LUAD (Chen et al., 2024).

### 3.2 Digestive system diseases

#### 3.2.1 Liver diseases

##### 3.2.1.1 Drug-induced liver injury (DILI) and acute liver failure (ALF)

The ferroptosis–HMGB1 axis plays a critical role in DILI, notably under antibiotic and chemotherapeutic exposure. Ceftiofur, a common veterinary antibiotic, enhanced hepatic ferroptosis evidenced by upregulation of key markers, including TfR1, ACSL4, cyclooxygenase-2 (COX2), HMGB1, and thioredoxin-interacting protein (TXNIP), along with downregulation of protective factors such as GPX4, SLC7A11, and ferritin (Hou L. et al., 2024). Similarly, chemotherapeutics such as methotrexate (MTX) trigger HMGB1-mediated autophagic ferroptosis via NCOA4-dependent ferritinophagy, leading to iron overload and lipid peroxidation. The inhibition of HMGB1 by glycyrrhizic acid (GA) effectively mitigates MTX-induced liver injury, highlighting its therapeutic potential (Wang et al., 2024b). Furthermore, in ALF, oxidative stress and ferroptosis are key drivers of hepatocyte damage. Glycyrrhizin (GLY), a verified selective inhibitor of HMGB1, attenuates ALF by reducing HMGB1 expression, restoring GPX4 and GSH, and activating Nrf2/HO-1 signaling, thereby breaking the feedback loop between HMGB1 release and oxidative damage, underscoring its therapeutic value in ALF (Wang et al., 2019).

##### 3.2.1.2 Hepatic ischemia-reperfusion injury (HIRI)

HIRI involves the HMGB1-ferroptosis axis that amplifies inflammation and tissue damage. In macrophages, Caspase 6 promotes HMGB1 secretion through the receptor-interacting serine/threonine-protein kinase 1 (RIPK1)-kinase 1 (ASK1) signaling pathway, enhancing hepatocyte ferroptosis and establishing a feed-forward injury loop (Lin et al., 2023). In parallel, ischemia-induced PCBP2 downregulation promotes oxidative stress and HIF1α activation via p300, leading to HMGB1 translocation and subsequent M1 macrophage recruitment. Acteoside, a natural compound, restores PCBP2 expression and system Xc^−^ integrity, thereby limiting HMGB1-mediated inflammation in HIRI (Jia et al., 2025). Together, these findings highlight that HMGB1 contributes to ferroptosis-driven inflammation in HIRI through distinct pathways, providing multiple targets for intervention.

##### 3.2.1.3 Hepatic fibrosis (HF)

Hepatic fibrosis is marked by activated hepatic stellate cells (HSCs) and extracellular matrix (ECM) accumulation. Inducing ferroptosis in HSCs represents a potential antifibrotic strategy. Celastrol, a bioactive triterpenoid, induces ferroptosis in HSCs by disrupting redox balance through inhibition of PRDXs and upregulation of HO-1, promoting lipid peroxidation and iron overload. *In vivo*, celastrol alleviated CCl_4_-induced fibrosis, which was reversed by ferroptosis inhibitors. Although HMGB1 is released from ferroptotic HSCs and may act as a DAMP to propagate inflammation and fibrosis, its precise role in celastrol-induced ferroptosis requires further investigation (Luo et al., 2022).

##### 3.2.1.4 Hepatocellular carcinoma (HCC)

In hepatocellular carcinoma (HCC), the ferroptosis–HMGB1 axis exhibits dual and context-dependent effects. On one hand, ferroptosis may trigger immunogenic cell death (ICD) and CD8^+^ T cell recruitment; on the other, HMGB1 released from ferroptotic cells promotes the accumulation of immunosuppressive myeloid-derived suppressor cells (MDSCs) and enhances programmed death-ligand 1 (PD-L1) expression, facilitating immune escape (Conche et al., 2023). Moreover, under hypoxia, cytoplasmic HMGB1 suppresses ferroptosis by enhancing GPX4 and HSPB1 expression, promoting tumor survival (He et al., 2017). Overall, the dual role underscores the complexity of targeting ferroptosis in HCC. Targeting the ferroptosis-HMGB1 axis, particularly in combination with immune checkpoint blockade (ICB), may help overcome resistance and improve therapeutic efficacy.

#### 3.2.2 Esophageal adenocarcinoma (EAC)

HMGB1 was identified as a key ferroptosis-related prognostic marker in esophageal adenocarcinoma (EAC). Through bioinformatic screening and Cox regression analysis, HMGB1 was identified among four ferroptosis-related genes (alongside *ATF3*, *ATM*, and *ATG5*) as independent prognostic markers in EAC. High HMGB1 levels correlated with poor survival, highlighting its potential as a negative prognostic biomarker. HMGB1 expression promotes ferroptosis via oxidative stress pathways and inhibits Nrf2 antioxidant responses, forming a vicious cycle of oxidative damage. Conversely, its suppression reduces ROS and ferroptotic damage (Chen F. et al., 2022).

#### 3.2.3 Pancreatic diseases

##### 3.2.3.1 Acute pancreatitis (AP)

Ferroptosis contributes to severe acute pancreatitis (AP) via DAMP release, especially HMGB1. The classical Chinese medicine formula Da Cheng Qi Decoction suppressed NADPH oxidase 2 (NOX2)/GPX4 signaling-mediated ferroptosis and HMGB1 release, reducing pancreatic inflammation (Chen et al., 2025). Pancreatic-specific deletion of the circadian transcription factor ARNTL led to enhanced susceptibility to L-arginine-induced AP, with increased mortality, neutrophil infiltration, and HMGB1 release. Mechanistically, ARNTL maintained key anti-ferroptotic regulators, including SLC7A11, GPX4, and Nrf2. Importantly, ferroptosis inhibition or HMGB1 neutralization effectively attenuated AP in ARNTL-deficient mice, supporting a causal role for ferroptosis-induced HMGB1 release in sterile pancreatic inflammation (Liu et al., 2020).

##### 3.2.3.2 Pancreatic ductal adenocarcinoma (PDAC)

The role of ferroptosis and HMGB1 is context-dependent in pancreatic ductal adenocarcinoma (PDAC). In *Kras*-mutant PDAC models, ferroptosis triggered by dietary iron overload or GPX4 depletion leads to the release of oxidized nucleobase 8-hydroxyguanosine (8-OHG), activating the *TMEM173*/stimulator of interferon genes (STING)-dependent macrophage inflammation via IL-6/nitric oxide synthase 2 (NOS2) (Dai et al., 2020). Conversely, under specific therapeutic contexts, controlled induction of ferroptosis can unleash HMGB1 as an immunogenic signal to stimulate antitumor immunity. A novel ferroptosis inducer, N6F11, was shown to selectively degrade GPX4 in tumor cells via *TRIM25*-mediated ubiquitination and activate HMGB1-dependent CD8^+^ T cells, enhancing ICB efficacy in *Kras/Trp53*-mutant PDAC models (Jingbo Li et al., 2023). Additionally, pirin (PIR) overexpression sequesters HMGB1 in the nucleus and suppresses BECN1–ACSL4-mediated autophagic ferroptosis, whereas PIR knockdown promotes HMGB1 cytosolic translocation and ferroptosis (Hu et al., 2021). Macrophage capping protein (MCP) inhibition reduces PIR ufmylation and GPX4 transcription, triggering ferroptosis and HMGB1 release, which promotes M1-like macrophage polarization and anti-tumor immunity, highlighting the therapeutic relevance of targeting this pathway (Li et al., 2024).

#### 3.2.4 Colorectal diseases

##### 3.2.4.1 Ulcerative colitis (UC)

Oxymatrine (OY), a major alkaloid extracted from *Sophora flavescens*, has shown efficacy in ameliorating DSS-induced Ulcerative Colitis (UC) *in vivo*. Bioinformatics and experimental evidence revealed that OY modulates multiple targets involved in ferroptosis and inflammation. Specifically, OY significantly reduced levels of ferroptosis markers such as Fe^2+^ accumulation and GSH depletion, along with downregulating inflammatory mediators including IL-1β, IL-6, TNF-α, HMGB1, and NLRP3. At the transcriptional and protein levels, OY also suppressed the expression of several hub genes linked to ferroptosis–inflammation synergy, including *NOS2*, *HIF1A*, *IDO1*, *DUOX2*, and *TIMP1* (Gao et al., 2024).

##### 3.2.4.2 Colorectal cancer (CRC)

Bioinformatics screening across multiple datasets identified five key ferroptosis-related genes, including *ELAVL1*, *GPX2*, *EPAS1*, *SLC7A5*, and *HMGB1*, with significant dysregulation in CRC tissues. Among them, *HMGB1* expression was notably elevated in tumor samples compared to healthy controls, suggesting its role in tumorigenesis and ferroptosis resistance. Given HMGB1’s dual capacity to drive inflammation and modulate redox signaling, its upregulation may confer tumor-promoting effects by evading ferroptotic death. These findings underscore the importance of the HMGB1–ferroptosis axis in CRC progression and offer a rationale for targeting HMGB1 or associated ferroptosis regulators (e.g., *GPX2*, *SLC7A5*) in CRC therapy (Huang et al., 2022).

#### 3.2.5 Small intestinal injury

Chronic exposure to hexavalent chromium [Cr (VI)] induced ferroptosis-driven intestinal injury. In broilers, chronic Cr (VI) exposure disrupted intestinal architecture, tight junctions (ZO-1, Occludin, Claudin-1), and gut microbiota, leading to barrier dysfunction. Mechanistically, Cr (VI) triggered ferroptosis as evidenced by increased Fe^2+^, ROS, and LPO levels, along with downregulation of GPX4 and SLC7A11. HMGB1 was subsequently released and activated the RAGE-p38-MAPK pathway, which upregulated TfR1 and exacerbated intracellular iron accumulation. This feed-forward loop amplified oxidative stress and ferroptosis, contributing to intestinal barrier dysfunction and impaired nutrient absorption (Wang J. et al., 2025).

#### 3.2.6 Chemotherapy-induced nausea and vomiting (CINV)

Cisplatin, a commonly used chemotherapeutic agent, induces gastrointestinal inflammation and mucosal injury, in part by promoting cell ferroptosis in the gastrointestinal tract. Xiao-Ban-Xia decoction, a traditional Chinese herbal formula, reduced iron overload, oxidative stress, and HMGB1 levels, while activating Nrf2/SLC7A11/GPX4 signaling to suppress ferroptosis and preserve mucosal integrity (Liang et al., 2024). Targeting the ferroptosis–HMGB1 axis may thus offer a novel strategy for managing CINV.

### 3.3 Nervous system diseases

#### 3.3.1 Acute brain injury

HMGB1 plays a central role in orchestrating ferroptosis through diverse yet overlapping signaling pathways in various forms of acute brain injury, including ischemic, hypoxic, and traumatic insults.

In ischemic stroke models such as transient middle cerebral artery occlusion (tMCAO), HMGB1 release promotes neuronal and microglial damage through multiple mechanisms. Glycyrrhizic acid (GA) suppressed HMGB1 and downstream alpha kinase 1 (ALPK1), attenuated microglial pyroptosis and ferroptosis by suppressing the ALPK1/NF-κB/NLRP3/gasdermin D (GSDMD) and janus kinase 2 (JAK2)/STAT3 signaling pathways, thereby reducing infarct volume and improving neurological function (Du et al., 2025). Furthermore, HMGB1 released from necrotic neurons binds to astrocytic TLR4 and C-X-C chemokine receptor type 4 (CXCR4), inducing hepcidin-mediated ferroportin degradation, neuronal iron overload, and exacerbated ferroptosis (Davaanyam et al., 2023). Ischemic stroke also triggers secondary liver injury, wherein HMGB1 activates hepatic TLR4 and RAGE signaling, amplifying systemic hepcidin expression and brain damage, which could be reversed by HMGB1-neutralizing antibodies or A-box fragments (Davaanyam et al., 2024). In neonatal hypoxic–ischemic brain damage (HIBD), the HMGB1 inhibitor GLY alleviates neuronal injury by restoring GPX4 levels, reducing ACSL4 and prostaglandin-endoperoxide synthase 2 (PTGS2) expression, and reversing ferroptotic ultrastructural changes (Zhu et al., 2022). Similarly, in LPS-treated hippocampal HT22 cells, esketamine, a non-competitive NMDA receptor antagonist, downregulated HMGB1, reduces ROS/Fe^2+^/LPO, and inhibited ACSL4, PTGS2, TfR1, and FPN, thereby mitigating ferroptosis (Wang J. et al., 2022). The Nrf2 signaling pathway also emerges as a key downstream effector regulated by HMGB1. In traumatic brain injury (TBI), treatment with Annexin A5 (A5) alleviates cerebral edema, blood–brain barrier (BBB) disruption, neuronal death, and iron accumulation. Mechanistically, A5 suppresses HMGB1/NF-κB signaling, activates the Nrf2/HO-1 pathway, promotes anti-inflammatory M2 microglial polarization, and limits peripheral immune infiltration, collectively reducing ferroptosis and secondary injury (Gao et al., 2023). In hemorrhagic transformation (HT) after thrombolysis, lipocalin-2 (LCN2) promotes endothelial ferroptosis via the HMGB1/Nrf2/HO-1 axis, increasing hemorrhagic risk, whereas LCN2 inhibition preserves blood–brain barrier integrity (Liu J. et al., 2024). Additionally, in intracerebral hemorrhage (ICH), the flavanol (−)-epicatechin (EC) activates both Nrf2-dependent and -independent pathways, indirectly suppressing HMGB1-mediated ferroptosis and reducing neuronal degeneration (Chang et al., 2014).

#### 3.3.2 Neurodegenerative diseases

##### 3.3.2.1 Alzheimer’s disease (AD) and Parkinson’s disease (PD)

Ferroptosis is increasingly implicated in the progression of Alzheimer’s disease (AD) and Parkinson’s disease (PD) (Homma et al., 2024). In PD, elevated HMGB1, ceruloplasmin, and transferrin in dopaminergic neurons contribute to ferroptosis and neuroinflammation. Anti-HMGB1 therapy attenuates these effects by blocking extracellular HMGB1-mediated activation of NF-κB and JAK2/STAT3 pathways (Liu et al., 2023). In AD, ferroptotic stress driven by amyloid-β (Aβ) plaques and tau pathology activates HMGB1 and triggers microglial ferroptosis (Homma et al., 2024). Intranasal delivery of amino-functionalized graphene quantum dots (A-GQDs) induce hippocampal ferroptosis. This process increases TNF-α secretion via HMGB1 release and promotes p38 MAPK-mediated YAP nuclear translocation, transcriptionally activating *TFRC* and *ALOXE3* to enhance iron accumulation and lipid peroxidation (Wu et al., 2023).

#### 3.3.3 Glioblastoma (GBM)

Glioblastoma (GBM) is an aggressive primary brain tumor that exhibits resistance to conventional therapies. Ferroptosis-inducing strategies represent promising alternatives. Auranofin (AF), a thioredoxin reductase 1 (TrxR1) inhibitor, combined with cold atmospheric plasma-treated PBS, induces ferroptosis in GBM cells by disrupting redox homeostasis, leading to LPO and intracellular ROS accumulation. This combination markedly reduces TrxR activity and GSH levels, promoting the release of HMGB1 and other DAMPs, which promote DC maturation and enhance immunogenicity. These ferroptosis-associated changes collectively suppress tumor growth and prolong survival *in vivo* (Jinthe et al., 2021). In sonodynamic therapy, boron nitride nanoparticles with chlorin e6 (BNPD-Ce6) delivered via platelets induce quasi-immunogenic ferroptosis in GBM cells, which release ATP/CRT/HSP90 but lack HMGB1. Platelet-derived HMGB1 compensates for this deficiency, re-establishing immunogenicity and amplifying ferroptosis-mediated tumor suppression (Wang M. et al., 2025).

#### 3.3.4 Spinal cord injury (SCI)

Spinal cord injury (SCI) involves primary mechanical trauma followed by secondary injuries, including oxidative stress, inflammation, and ferroptosis. Damaged neurons and glial cells release HMGB1 into the extracellular space, cerebrospinal fluid, and bloodstream, exacerbating injury by disrupting the BBB and triggering neuroinflammation (Wu and Li, 2023). SYVN1, an E3 ubiquitin ligase, binds HMGB1 and promotes its ubiquitination-dependent degradation, suppressing ferroptosis and activates Nrf2/HO-1 signaling in spinal neurons under ischemia-reperfusion conditions (Lili Guo et al., 2023). Similarly, neutral polysaccharide from *Gastrodia elata* also reduces ferroptosis and neuroinflammation by elevating GPX4, activating Nrf2/HO-1, and lowering ROS and proinflammatory cytokines (Zhang et al., 2024). Targeting the HMGB1/Nrf2/HO-1 axis may offer a promising strategy for mitigating ferroptosis-related damage in SCI.

### 3.4 Circulatory system diseases

#### 3.4.1 Vascular diseases

##### 3.4.1.1 Ischemia-reperfusion injury (IRI)

Ischemia-reperfusion injury (IRI) is a major pathological event in vascular diseases such as stroke and myocardial infarction, contributing substantially to tissue damage following the restoration of blood flow. Ferroptosis-driven HMGB1 release has been implicated in myocardial and cerebral IRI, linking redox imbalance to inflammation. In myocardial IRI, pharmacological inhibition of HMGB1 using GLY markedly reduces proinflammatory cytokines (e.g., TNF-α, IL-1β, IL-6) and histopathological damage, while restoring GPX4 expression and antioxidant capacity. Similar protective effects are observed with TAK-242, a TLR4 antagonist, reinforcing the functional relevance of the HMGB1-TLR4-GPX4 signaling cascade in regulating ferroptosis (Zhu et al., 2024). Moreover, fragmented oxidized phosphatidylcholines (OxPCs), including POVPC and PONPC, exacerbate myocardial ferroptosis by impairing mitochondrial respiration, disrupting calcium homeostasis, and suppressing GPX4, although these effects may occur independently of HMGB1 involvement (Stamenkovic et al., 2021). In cerebral IRI models, ferroptosis inhibitors (Fer-1, AA9) alleviate neurological injury by enhancing GPX4 and Nrf2/HO-1 signaling while suppressing HMGB1 and NF-κB activation (Chen Y. et al., 2023).

##### 3.4.1.2 Abdominal aortic aneurysms (AAA)

Abdominal aortic aneurysms (AAA) are characterized by chronic vascular inflammation, smooth muscle cell loss, and ECM degradation. Ferroptosis contributes to smooth muscle cell death and triggers the release of HMGB1, which in turn activates inflammatory signaling via NOX2. Resolvin D1 (RvD1), a specialized pro-resolving lipid mediator derived from docosahexaenoic acid, has been identified as a potent inhibitor of both inflammation and ferroptosis in AAA. Acting through FPR2, RvD1 suppresses NOX2-dependent ROS generation, inhibits HMGB1 release, and attenuates vascular inflammation and remodeling. In addition, RvD1 promotes M2 macrophage polarization and enhances efferocytosis, thereby disrupting the ferroptosis–HMGB1–inflammation feedback loop (Amanda et al., 2022).

##### 3.4.1.3 Vascular endothelial dysfunction

Endothelial dysfunction, a hallmark of cardiovascular disease, is exacerbated by ferroptosis and HMGB1-driven inflammation. In human umbilical vein endothelial cells (HUVECs), N-methyl-D-aspartate receptors (NMDARs) activation induces ferroptosis through the PP2A-AMPK-HMGB1 pathway, leading to increased lipid ROS, MDA, PTGS2, and decreased GPX4/GSH (Han et al., 2024). Likewise, LPS-stimulated HUVECs show ferroptosis and HMGB1 upregulation, which are suppressed by Fer-1. Nrf2 activation counteracts these effects by upregulating GPX4, inhibiting ferritinophagy, and reducing NOX4-mediated stress (Hou H. et al., 2024).

##### 3.4.1.4 Valvular atrial fibrillation (VAF)

Valvular atrial fibrillation (VAF) is a common and prognostically adverse complication of valvular heart disease, characterized by chronic inflammation and progressive atrial fibrosis. Transcriptomic analysis of cardiac tissues from VAF patients, compared to those with valvular sinus rhythm, revealed significant upregulation of ferroptosis- and immunity-related genes, including *HMGB1*, highlighting its potential role as a key mediator linking ferroptosis to immune activation. Natural compounds such as curcumin and RSV, predicted to target *HMGB1* and *TGFBR1*, have demonstrated anti-ferroptosis and anti-inflammatory properties and may attenuate HMGB1-mediated responses in VAF. While preliminary evidence supports the involvement of the ferroptosis–HMGB1 axis in VAF progression, further experimental validation is warranted (Jiang et al., 2022).

#### 3.4.2 Cardiac diseases

##### 3.4.2.1 Drug-induced cardiomyopathy

Drug-induced cardiomyopathy, particularly caused by chemotherapeutic agents such as doxorubicin (DOX) and sorafenib, remains a serious complication of cancer therapy and is closely associated with ferroptosis and HMGB1-mediated inflammation (Liu C. et al., 2024; Zhang et al., 2021). Sorafenib upregulated sodium/glucose cotransporter 2 (SGLT2) in cardiomyocytes, promoting glucose and sodium influx, calcium overload, and ultimately cell ferroptosis. This is accompanied by NF-κB activation and the release of HMGB1 and other DAMPs, which drive inflammatory responses and macrophage recruitment. SGLT2 inhibitors such as empagliflozin and dapagliflozin counteract these effects by restoring GPX4 and SLC7A11 expression, inhibiting NF-κB phosphorylation, and reducing HMGB1 release (Liu C. et al., 2024). Similarly, DOX induces cardiotoxicity via GPX4 depletion and lipid peroxidation (MDA, PTGS2), which is exacerbated by HMGB1 overexpression and attenuated by HMGB1 silencing or ferroptosis inhibitors (Fer-1, DXZ) (Zhang et al., 2021). DOX also promotes the formation of NETs, which exacerbate ferroptosis via the HMGB1/TLR4/YAP signaling axis (Zhao P. et al., 2024).

##### 3.4.2.2 Endotoxemia-associated cardiac injury

Endotoxemia, often induced by LPS, causes sepsis-related myocardial injury characterized by contractile dysfunction, oxidative stress, and inflammatory cell death. In both *in vivo* and *in vitro* models of LPS-induced endotoxemia, RSV significantly alleviates myocardial ferroptosis and injury. This cardioprotective effect is mediated through upregulation of miR-149, which directly targets HMGB1 and suppresses its expression. Notably, HMGB1 overexpression abolishes the protective effects of RSV, confirming the functional role of the miR-149/HMGB1 axis in regulating inflammation-driven ferroptosis in septic cardiomyopathy (Wang X. et al., 2022).

#### 3.4.3 Hematologic malignancies

Hematologic malignancies, including chronic lymphocytic leukemia (CLL), chronic myeloid leukemia (CML), acute myeloid leukemia (AML), promyelocytic leukemia (PML), and multiple myeloma (MM), represent a diverse group of cancers arising from the hematopoietic and lymphoid tissues. These malignancies are often characterized by complex molecular pathogenesis and frequent resistance to chemotherapy.

CLL exemplifies the immunogenic potential of ferroptosis, where treatment with the spirocyclic dimer SpiD3 induces ferroptosis and ICD, leading to the release of classical DAMPs such as CRT, ATP, and HMGB1. HMGB1 secretion, in particular, plays a pivotal role in promoting bone marrow-derived DC recruitment and activation, enhancing antigen presentation and T cell–mediated anti-leukemia responses (Schmitz et al., 2024). Selinexor-resistant CML exhibits a ferroptosis-suppressive phenotype. Transcriptomic analysis revealed upregulation of ferroptosis-suppressive molecules (FTH1, SLC7A11) and downregulation of ferroptosis-inducing factors, including HMGB1 and MTDH. This shift promotes leukemic stemness and drug evasion. Notably, co-treatment with the ferroptosis inducer RSL3 restores ferroptotic sensitivity and overcomes selinexor resistance *in vitro*, highlighting the therapeutic potential of reactivating the ferroptosis–HMGB1 axis in resistant CML (Sun et al., 2023). AML shares similar ferroptosis-dependent vulnerabilities. The circadian regulator BMAL1 promotes chemoresistance by suppressing ferroptosis through the BMAL1-HMGB1-GPX4 axis. Knockdown of BMAL1 reduces HMGB1 expression, enhances ferroptosis, and sensitizes AML cells to standard therapies (Hong Zheng et al., 2024). Additionally, SIRT1 mediates cytarabine resistance by promoting HMGB1 translocation and ACSL4 expression, thereby inhibiting ferroptosis. Targeting the SIRT1–HMGB1 pathway restores ferroptotic sensitivity and attenuates tumor growth *in vivo* (Kong et al., 2025). In NRAS-mutant AML, HMGB1 regulates ferroptosis via the RAS–JNK/p38 pathway, further supporting its role as a ferroptosis mediator (Ye et al., 2019). In PML, arsenic trioxide (ATO) induces ferroptosis and ICD in an HMGB1/ACSL4-dependent manner (Kepp et al., 2023). In MM, shikonin triggers ferroptosis via GOT1-mediated ferritinophagy and HMGB1 release (Li et al., 2022). Collectively, these findings underscore the central role of the ferroptosis-HMGB1 axis in regulating cell death, immune activation, and therapeutic responsiveness across diverse hematologic malignancies.

### 3.5 Urinary system diseases

#### 3.5.1 Renal tubular dysfunction

Renal tubular injury, arising from diverse etiologies, frequently involves the ferroptosis-HMGB1 axis as a central mechanism. In transfusion-dependent β-thalassemia (β-TM), iron overload and gut metabolite TMAO promote renal dysfunction by downregulating SIRT1, inducing HMGB1 release, and upregulating the ferroptosis marker ACSL4 (Asmaa et al., 2024). Further supporting this axis, Zhao et al. demonstrated that cytosolic HMGB1 binds directly to ACSL4 in renal tubular epithelial cells following ischemia–reperfusion injury, initiating ferroptosis and tubular injury. Both genetic deletion and pharmacological inhibition of HMGB1 nuclear export markedly reduced ferroptosis and renal damage (Zhao et al., 2023a).

#### 3.5.2 Acute kidney injury (AKI)

Acute kidney injury (AKI), affecting up to 50% of critically ill patients, arises from diverse etiologies such as ischemia/reperfusion (I/R), sepsis, nephrotoxins (e.g., cisplatin), crush syndrome, and environmental exposures. Despite this etiological heterogeneity, increasing evidence supports the ferroptosis-HMGB1 axis as a core unifying mechanism that orchestrates oxidative stress, inflammation, and tubular epithelial cell death across multiple AKI subtypes (Deng Z. et al., 2024; Zhang et al., 2023; Xue et al., 2021).

By activating RAGE/TLR4–NF-κB and suppressing the Nrf2–GPX4/SLC7A11 pathway, HMGB1 establishes a feedforward loop that amplifies inflammation and ferroptosis in AKI (Qiao et al., 2023). This central mechanism has been demonstrated in various AKI models. In cisplatin-induced AKI, upregulation of *TACSTD2* in renal tissues enhanced ferroptosis cell death and tubular damage via HMGB1-dependent TLR4/NF-κB activation (Deng Z. et al., 2024), whereas the Chinese herbal formulation Shenshuaifu granule alleviates damage by modulating the p53/SLC7A11/GPX4 pathway and reducing HMGB1 expression (Jin et al., 2023). In crush syndrome–associated AKI (CS-AKI), massive HMGB1 release during rhabdomyolysis exacerbates inflammation through RAGE/TLR4 activation and aggravates ferroptosis via Nrf2 pathway inhibition and ACSL4 upregulation. Ferroptotic tubular cells further release extracellular double-stranded DNA (dsDNA), which activates the cGAS-STING pathway and induces ATF3, a transcriptional repressor of SLC7A11, thus deepening ferroptotic injury. In addition, platelet-primed macrophages release macrophage extracellular traps rich in HMGB1 and histones, which directly impair mitochondrial function and trigger LPO (Qiao et al., 2023). Environmental co-exposure to cadmium and polystyrene nanoplastics induces ferroptosis and mitophagy, characterized by upregulation of SLC7A11, GPX4, HMGB1, and PTGS2, as well as mitophagy markers like BECN1 and Microtubule-associated protein 1A/1B-light chain 3 (LC3). Here, HMGB1-RAGE/TLR4 signaling acts as a downstream amplifier of ferroptosis-driven inflammation (Qiu et al., 2023). In sepsis-associated AKI induced by LPS, ferritinophagy-triggered ferroptosis contributes to HMGB1 release and mitochondrial damage, while natural compounds such as isoliquiritigenin restore GPX4/SLC7A11 levels and inhibit HMGB1 secretion, offering protective effects (Tang et al., 2021). Similarly, the neonicotinoid pesticide imidacloprid (IMI) has been shown to induce ferroptosis in renal tissues, leading to LPO and Nrf2 suppression, and triggering the HMGB1-RAGE/TLR4-NF-κB inflammatory axis, which in turn promotes pyroptotic injury (Zhang et al., 2023). In the context of heat stroke-related AKI, elevated levels of Aifm2, HMGB1, and RAGE in kidney tissues suggest ferroptosis involvement, supported by increased synthesis of unsaturated fatty acids as a biochemical hallmark of LPO (Xue et al., 2021). Beyond acute injury, the ferroptosis-HMGB1 axis is also implicated in the progression from AKI to chronic kidney disease (CKD). Tubular epithelial cell–derived intracellular HMGB1 has been shown to sensitize regenerating tubular cells to oxidative stress, thereby impairing tissue repair and promoting fibrosis. Importantly, pharmacological or genetic inhibition of HMGB1 after the acute injury phase, using agents such as ethyl pyruvate or GA, can improve long-term renal outcomes without affecting the severity of acute injury, highlighting a potentially time-sensitive therapeutic window (Zhao et al., 2023b).

#### 3.5.3 Chronic kidney disease (CKD)

Chronic kidney disease (CKD), characterized by renal fibrosis, is driven by sustained ferroptosis and HMGB1-mediated inflammation. In multiple murine models of kidney fibrosis (e.g., UUO, adenine diet, I/R, 5/6 nephrectomy, folic acid), key ferroptotic features such as iron overload, oxidative stress, GPX4 downregulation, and ACSL4 upregulation are consistently observed. Pharmacological inhibition of ferroptosis using deferoxamine or Fer-1 markedly attenuates fibrosis (Wang et al., 2023a). The natural flavonol fisetin exerts renoprotection by targeting ACSL4, downregulating COX2, upregulating GPX4, and suppressing HMGB1-amplified inflammation, highlighting the ACSL4–ferroptosis–HMGB1 axis as a key fibrogenic pathway (Wang et al., 2023b).

#### 3.5.4 Calcium oxalate (CaOx) kidney stone

Calcium oxalate (CaOx) stones, the most common form of nephrolithiasis, cause tubular injury and inflammation through crystal adhesion and oxidative stress. Recent evidence implicates ferroptosis in CaOx-induced damage: crystal exposure leads to Fe^2+^ accumulation, LPO, and mitochondrial dysfunction in HK-2 cells, accompanied by downregulation of GPX4 and SLC7A11 and upregulation of ACSL4, ceruloplasmin, and transferrin. This is further exacerbated by activation of the Nrf2/HO-1 pathway, which paradoxically intensifies oxidative stress under sustained insult. Transcriptomic analysis identified *ANKRD1* as a key upstream driver, promoting ferroptosis via the p53/SLC7A11 axis. HMGB1, elevated in response to ferroptotic injury, may amplify inflammation and facilitate crystal deposition (Zhao et al., 2023c).

#### 3.5.5 Bladder cancer

Bladder cancer, a prevalent urinary tract malignancy, shows limited response to ICB. Recent studies highlight ferroptosis-induced ICD as a key enhancer of anti-tumor immunity and ICB efficacy. A mitochondrial-targeted liposomal system (BQR@MLipo), delivering the dihydroorotate dehydrogenase inhibitor brequinar, induces mitochondrial ferroptosis, HMGB1 and mtDNA release, and activates Cgas-STING signaling to promote DC maturation and CD8^+^ T cell infiltration, thereby boosting ICB responses (Ding et al., 2023). Separately, glutathione S-transferase zeta 1 (GSTZ1) overexpression triggers ferroptosis via HMGB1-GPX4 signaling by depleting GPX4 and GSH and increasing oxidative stress, suppressing tumor proliferation—a phenotype reversed by HMGB1 knockdown or GPX4 restoration (Zhu et al., 2023).

### 3.6 Locomotor system diseases

#### 3.6.1 Osteoarthritis (OA) and knee osteoarthritis (KOA)

Osteoarthritis (OA), especially knee osteoarthritis (KOA), is a common degenerative joint disease in the aging population. HMGB1 released from ferroptotic osteocytes promotes osteoclastogenesis through RAGE/TLR4/NF-κB signaling, driving bone resorption (Zhao et al., 2025). The traditional Chinese medicine Jianpi-Tongluo Formula confers cartilage-protective and anti-edema effects by downregulating NCOA4, HMGB1, and GSK3B, thereby mitigating oxidative stress and iron dysregulation (Liu Y. et al., 2024). In KOA, METTL3-mediated N^6^-methyladenosine (m^6^A) modification of HMGB1 accelerates chondrocyte ferroptosis and extracellular matrix degradation, while METTL3 knockout alleviates synovial inflammation, cartilage damage, and pain, effects reversed by HMGB1 overexpression (Bao et al., 2024).

#### 3.6.2 Lower limb ischemia/reperfusion injury

Lower limb ischemia/reperfusion (I/R) injury often causes severe skeletal muscle damage. Luo et al. (Fengdan Wang et al., 2023) showed that syringic acid (SA) protects against I/R-induced injury by suppressing HMGB1, activating the Nrf2/HO-1 pathway, and upregulating GPX4 and SLC7A11. SA reduced inflammation, Fe^2+^ accumulation, LPO, and apoptosis in both murine and C2C12 cell models. Notably, HMGB1 overexpression abolished the protective effects of SA, confirming its pivotal role in ferroptosis-mediated muscle damage and positioning SA as a potential therapeutic agent.

#### 3.6.3 Ischemic muscle satellite cell dysfunction in peripheral artery disease (PAD)

Peripheral artery disease (PAD) impairs skeletal muscle regeneration by disrupting muscle satellite cell (MuSC) function. Tran et al. identified a ferroptosis-associated transcriptional signature in ischemic MuSCs from PAD patients, marked by iron metabolism dysregulation, GPX4 downregulation, and elevated HMGB1 expression correlated with LPO (Tran et al., 2022). Fer-1 reversed ferroptotic damage *in vitro*, and iron/lipofuscin deposits were confirmed in PAD tissues. Further profiling showed reduced quiescence and increased myofibroblastic/apoptotic phenotypes, alongside upregulated ferroptosis-related genes and DAMPs like HMGB1 (Lillian Tran et al., 2023).

### 3.7 Endocrine system diseases

#### 3.7.1 Adrenocortical carcinoma (ACC)

Adrenocortical carcinoma (ACC) is a rare but aggressive malignancy with poor prognosis. Bioinformatic modeling has consistently identified HMGB1 as a core gene within ferroptosis-related prognostic signatures. Ye et al. constructed a six-gene model in which high HMGB1 expression, alongside SLC7A11 and ACSL4, was associated with poorer survival and reduced immune cell infiltration (Chen et al., 2021b). Lin et al. further confirmed HMGB1’s prognostic value within a broader 17-gene ferroptosis-based model that outperformed traditional TNM staging in predicting patient outcomes (Lin et al., 2022). These findings establish HMGB1 as both a prognostic biomarker and a therapeutic target in ACC.

#### 3.7.2 Diabetes

Diabetes mellitus is associated with systemic oxidative stress and inflammation, contributing to complications such as diabetic kidney disease, retinopathy, and liver injury. In gestational diabetes, Lv et al. identified 12 ferroptosis- and autophagy-related genes, including *HMGB1*, enriched in the PI3K-AKT signaling pathway and correlated with immune infiltration (Lv et al., 2024). For type 2 diabetes mellitus, elevated HMGB1 promotes platelet-derived microparticles and inflammatory markers (e.g., soluble CD40 ligand, E-selectin), both of which are reduced by combined SGLT2 and DPP-4 inhibitor treatment (Nomura et al., 2024). Regarding diabetic retinopathy, RSV protects retinal endothelial cells via the SIRT1/HMGB1 axis, restoring GPX4 and SLC7A11 (Pen et al., 2025). HMGB1 also contributes to ferroptosis in mesangial cells in diabetic kidney disease via TLR4/NF-κB signaling, while its silencing restores antioxidant defenses (Wu et al., 2021). Moreover, in diabetic liver injury, Fer-1 reversed HMGB1 elevation and restored Nrf2 and ferroptosis-related markers, ameliorating histological damage (Stancic et al., 2022). Overall, the ferroptosis-HMGB1 axis links oxidative stress to immune dysregulation across multiple diabetic complications, suggesting that HMGB1 and its upstream or downstream pathways represent promising therapeutic targets.

### 3.8 Reproductive system diseases

#### 3.8.1 Female reproductive system diseases

Emerging evidence underscores the contribution of the ferroptosis-HMGB1 axis to female reproductive disorders, where it orchestrates LPO-driven cell death, inflammation, and tumor progression in both malignant and pregnancy-related conditions.

Breast cancer remains one of the most common malignancies among women, with high mortality primarily due to metastasis and recurrence. Ferroptosis has demonstrated potential in suppressing tumor progression and enhancing sensitivity to chemotherapy and radiotherapy (Desterke et al., 2023; Shetake et al., 2024). Transcriptome-integrated text mining identified a ferroptosis–ECM remodeling gene signature predictive of poor prognosis, particularly in triple-negative breast cancer (TNBC). A molecular score derived from 11 ferroptosis/ECM-related genes, including *HMGB1*, *IL6*, *EGFR*, and *TLR4*, was associated with aggressive tumor characteristics. Functional studies confirmed that ferroptosis inducers such as erastin and RSL3 activated this signature in TNBC cells (MDA-MB-231), indicating that ferroptosis may reshape ECM dynamics and the tumor microenvironment (Desterke et al., 2023). Additionally, nanotechnology-based ferroptosis inducers like T-LMD, a cRGD-modified magnetoliposome delivering iron oxide nanoparticles and DOX, potentiated ferroptosis through iron/lipid metabolism pathways. This strategy triggered ICD through ferroptosis-mediated mitochondrial dysfunction and HMGB1 release (Shetake et al., 2024).

In pregnancy disorders, the HMGB1-ferroptosis axis contributes to placental dysfunction. In preeclampsia, caspase-6 in macrophages induces trophoblast ferroptosis via HMGB1-mediated GPX4 downregulation. Conversely, caspase-6 silencing in macrophages reduced HMGB1 release, restored GPX4 levels, mitigated ferroptosis *in vitro* and *in vivo*, and improved pregnancy outcome (Deng Y. et al., 2024). In spontaneous abortion, elevated HMGB1 and ACSL4 promote LPS-induced trophoblast ferroptosis, linking inflammation to pregnancy loss (Dong et al., 2025).

In ovarian cancer, ferroptosis resistance contributes to immune evasion. Jin et al. showed that oncogenic C-MYC suppresses NCOA4-mediated ferritinophagy and ferroptosis, thereby reducing ROS and HMGB1 release. This promotes tumor proliferation, migration, and immune escape, underscoring the dual role of HMGB1 in cell death and immunogenicity (Jin et al., 2022). In cervical cancer, radiotherapy alters levels of ferroptosis markers such as 4-HNE and GPX4, correlating with immune cell infiltration. Elevated HMGB1 may modulate the tumor immune microenvironment and impact therapeutic outcomes, though its prognostic relevance remains uncertain (Oltean et al., 2022).

#### 3.8.2 Male reproductive system diseases

Emerging evidence highlights the ferroptosis-HMGB1 axis as a key pathogenic driver in male reproductive system disorders, ranging from oxidative testicular damage to immune-regulated prostate cancer progression. This axis not only exacerbates redox imbalance but also modulates inflammatory and immune responses in the reproductive microenvironment. In prostate cancer, ferroptosis enhances immunotherapy efficacy. Kim et al. reported that ferumoxytol, an iron oxide nanoparticle, triggered ferroptosis in prostate cancer cells and enhanced NK cell cytotoxicity by increasing interferon-gamma (IFN-γ) secretion, degranulation, and UL16-binding protein (ULBP) expression. This was accompanied by HMGB1 and PD-L1 upregulation, suggesting that ferroptosis enhances immune activation while modulating checkpoint signals. Combined treatment reduced tumor burden *in vivo*, suggesting HMGB1-mediated ferroptosis sensitizes tumors to NK clearance (Kim et al., 2022). In testicular injury, excess zinc triggered ferroptosis in porcine testis and ZnSO_4_-treated cells, marked by reduced SLC7A11, GPX4, ferritin, and increased MDA, CD71, transferrin, and HMGB1. Zinc also activated mitophagy (PTEN-induced kinase 1 [PINK1], Parkin, LC3-II), and mitophagy inhibition alleviated ferroptosis, indicating a mitophagy–ferroptosis crosstalk mediated by HMGB1-driven redox imbalance (Li Q. et al., 2023). Additionally, the plant growth regulator chlormequat chloride (CCC) induced apoptosis, pyroptosis, and ferroptosis in Leydig cells. Fer-1 more effectively restored cell viability and reduced IL-1β and HMGB1 levels than caspase inhibitors. Moreover, overexpression of ferritin light chain attenuated CCC-induced ferroptosis, further implicating HMGB1 and lipid ROS in inflammation-driven androgenic dysfunction (Wang et al., 2024c).

### 3.9 Inflammatory and autoimmune diseases

#### 3.9.1 Infection and sepsis

Infection-associated inflammatory conditions such as sepsis, radiation injury, and UVB-induced dermatitis involve a complex interplay between ferroptotic cell death and DAMP signaling, particularly mediated by HMGB1. These interactions contribute to immune imbalance, persistent inflammation, and tissue injury.

Sepsis is characterized by uncontrolled inflammation followed by immunosuppression. In sepsis, the small GTPase Rab26 protects macrophages from ferroptosis by stabilizing GPX4. Extracellular HMGB1 downregulates Rab26 in late-stage sepsis, promoting an immunosuppressive phenotype and increasing ferroptosis susceptibility (Gong et al., 2024). Radiation injury initiates ferroptosis and the release of DAMPs such as HMGB1, ATP, and HSPs, contributing to both antitumor immune responses and collateral tissue damage. In acute radiation syndrome, excessive HMGB1 release exacerbates systemic inflammation and predisposes to sepsis, suggesting that targeting ferroptosis or neutralizing HMGB1 may help mitigate radiation-induced lethality (Mengqin Zhu et al., 2021; Satoshi Yamaga et al., 2024). Similarly, UVB exposure induces macroautophagy-dependent ferroptosis in keratinocytes by upregulating TfR1, a process further amplified by HMGB1. This positive feedback loop aggravates oxidative damage and skin inflammation. Treatment with Senkyunolide I disrupts this ferroptosis-HMGB1-TfR1 axis and alleviates UVB-induced photodermatitis *in vitro* and *in vivo* (Wei et al., 2024).

#### 3.9.2 Autoimmune and immune dysregulation disorders

Chronic autoimmune disorders, including rheumatoid arthritis (RA), systemic lupus erythematosus (SLE), and hemophilia A (HA), involve persistent immune imbalance and inflammation. Increasing evidence implicates ferroptosis and HMGB1 signaling in driving tissue injury and maladaptive immune responses in these conditions.

RA pathogenesis involves iron overload–induced ferroptosis in anti-inflammatory M2 macrophages, which release HMGB1 and disrupt the M1/M2 balance. This release activates TLR4/STAT3 signaling in M1 macrophages, amplifying pro-inflammatory cytokine production. Inhibition of ferroptosis (via Lip-1 or GPX4 overexpression) or blockade of TLR4 signaling reduces synovial inflammation, highlighting the HMGB1/TLR4/STAT3 axis as a therapeutic target (Feng et al., 2024). In SLE, elevated serum HMGB1 levels correlate with ferroptosis/necroptosis markers and disease activity, supporting its role as a diagnostic and prognostic biomarker (Zhao G. et al., 2024). In HA, elevated C-X-C motif chemokine ligand 13 (CXCL13), a chemoattractant for follicular helper T (TFH) cells, promotes expansion of TFH and germinal center B cells in inhibitor-positive patients. Concurrent increases in BAFF and HMGB1 further support a pro-immunogenic milieu. Mechanistically, CXCL13 induces ferroptosis in endothelial cells by suppressing SLC7A11/GPX4, activating Nrf2, and generating ROS. HMGB1 released during this process enhances inflammatory signaling and B cell activation, implicating the CXCL13–ferroptosis–HMGB1 axis in inhibitor development and offering a new immunomodulatory target for HA (Luo et al., 2024).

#### 3.9.3 Skin and mucocutaneous inflammatory syndromes

The skin and mucosal surfaces serve as critical immunological barriers yet are highly vulnerable to ferroptosis-related injury under environmental or pathological stress. Ferroptosis-driven HMGB1 release has emerged as a key mediator of cutaneous inflammation, particularly in UV-induced dermatitis, drug reactions, and IRI. In UVB-triggered dermatitis, phospholipid peroxidation and GSH depletion lead to ferroptosis in keratinocytes, which release HMGB1 and initiate necroinflammatory responses. Inhibition of ferroptosis suppresses HMGB1 and alleviates inflammation more effectively than blocking apoptosis or pyroptosis, establishing ferroptosis as a primary driver in this setting (Vats et al., 2021). In Stevens-Johnson syndrome/toxic epidermal necrolysis (SJS/TEN), reduced SLC7A11/GPX4/GSH and elevated HMGB1 correlate with disease severity, ferroptosis inhibition restores antioxidant defenses and reduces cell death (Lin et al., 2024). In I/R-compromised skin flaps, ferroptosis contributes to tissue necrosis. Kaempferol improves flap survival by modulating SIRT1-HMGB1/TLR4-NF-κB and Nrf2-SLC7A11-GPX4 pathways. This results in reduced neutrophil infiltration, preserved mitochondrial function, and suppression of HMGB1 release, supporting antioxidant-mediated ferroptosis inhibition as a promising intervention (Wang A. et al., 2025).

**FIGURE 2:**
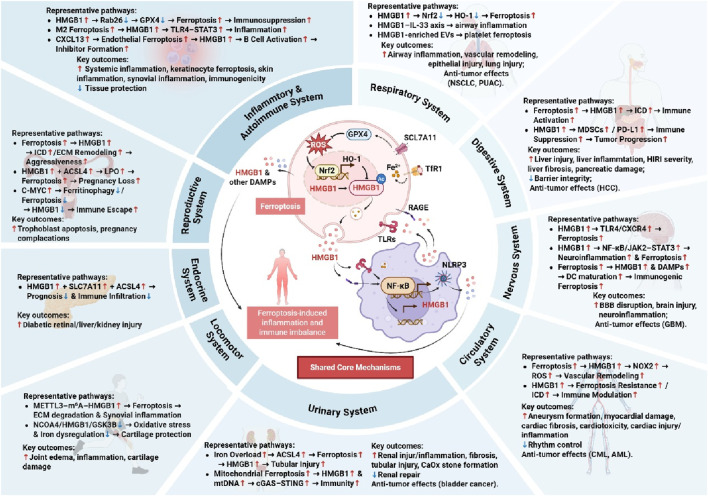
Integrated Pathways of Ferroptosis and HMGB1 in Systemic Diseases. HMGB1 promotes or modulates ferroptosis through various molecular intermediates, contributing to inflammation, tissue injury, immune activation, or suppression. Key outcomes include both detrimental effects and protective or anti-tumor responses, highlighting the dual role of HMGB1 and ferroptosis in different pathological conditions.

In summary, the ferroptosis-HMGB1 axis constitutes a unifying pathological mechanism across diverse systemic diseases, linking lipid peroxidation (LPO) to immune dysregulation and cellular injury. Despite disease-specific contexts, several upstream and downstream regulators consistently emerge as critical modulators of this axis. An expanding repertoire of therapeutic agents—from natural compounds to targeted inhibitors-has shown promise in modulating HMGB1-mediated ferroptosis across multiple organ systems. Table 1 summarizes core upstream and downstream proteins involved in HMGB1-regulated ferroptosis, and Figure 2 provides a visual overview of the ferroptosis–HMGB1 mechanisms underlying systemic pathologies.

**TABLE 1:** Summary of key upstream and downstream regulatory proteins involved in HMGB1-Mediated ferroptosis.

Target	Role	Mechanisms or pathways involved	Associated diseases	References
Nrf2	Upstream	Inhibits HMGB1 via antioxidant pathways and PIR-dependent suppression	TBI, ICH, AKI	Hu et al. (2021), Gao et al. (2023), Chang et al. (2014), Qiao et al. (2023)
PIR	Upstream	Inhibits HMGB1 and stabilizes Nrf2 to reduce ferroptosis	Pancreatic cancer	Hu et al. (2021), Li et al. (2024)
PCBP2	Upstream	Stabilizes SLC7A11 mRNA, inhibits HIF1α/p300 and HMGB1 release	HIRI	Jia et al. (2025)
NCOA4	Downstream	Mediates ferritinophagy, promotes Fe^2+^ release and ferroptosis	OC, DILI, AKI	Wang et al. (2024b), Tang et al. (2021), Jin et al. (2022)
BECN1	Downstream	Binds SLC7A11 to inhibit system Xc^−^, enhances autophagy and ferroptosis	NSCLC	Gechang et al. (2024)
YAP	Downstream	Activates *TFRC*, *ALOXE3* expression, enhances iron uptake and LPO.	AD	Wu et al. (2023)
TFRC	Downstream	Iron importer upregulated by YAP and HMGB1, promotes ferroptosis	AD	Wu et al. (2023)
GPX4	Downstream	Suppressed by HMGB1 to facilitate ferroptotic cascade	NSCLC, ALI, AKI, HCC	Gechang et al. (2024), Tang et al. (2021), Jia et al. (2024), Conche et al. (2023)
xCT	Downstream	Cystine/glutamate antiporter; central regulator of ferroptosis; HMGB1 suppresses its expression	NSCLC, ALI, AKI, HCC	Gechang et al. (2024), Tang et al. (2021), Jia et al. (2024), Conche et al. (2023)
HSPB1	Downstream	Modulated by HMGB1-Nrf2 axis to maintain mitochondrial integrity	ALI	Jia et al. (2024)

## 4 Therapeutic perspectives targeting HMGB1-Ferroptosis axis

Given its multifaceted involvement in redox imbalance, inflammation, and immunogenic cell death across systemic diseases, the HMGB1–ferroptosis axis has emerged as a promising therapeutic target. Preclinical data across respiratory, digestive, nervous, circulatory, and immune-related diseases suggest that therapeutically targeting this axis may yield substantial benefit. Current strategies can be broadly categorized into three approaches: (1) direct inhibition of HMGB1 to block its release or signaling via TLR4/RAGE; (2) modulation of ferroptosis to prevent iron-dependent oxidative cell death and secondary HMGB1 release; and (3) combined multi-target strategies involving nanocarriers, immune checkpoint inhibitors (ICIs), and natural products that simultaneously regulate ferroptosis and HMGB1-mediated inflammation.

### 4.1 Clinically advanced modulators

Several compounds with strong preclinical support have demonstrated promising effects in modulating the HMGB1–ferroptosis axis. Representative examples include: GLY, a natural triterpenoid, directly binds to HMGB1, blocks its extracellular release, and activates the Nrf2–HO-1–GPX4 axis. In models of ALF (Wang et al., 2019), HIBD (Zhu et al., 2022), and myocardial IRI (Zhu et al., 2024), GLY reduces ferroptosis markers, alleviates oxidative stress, and improves organ function. Fer-1, a synthetic ferroptosis inhibitor, suppresses LPO and restores redox homeostasis. Fer-1 alleviates tissue damage and inflammatory injury in diverse models such as allergic asthma (Li Y. et al., 2023), PH (Xie et al., 2022), cerebral IRI (Chen Y. et al., 2023), DOX-induced cardiomyopathy (Zhang et al., 2021), and diabetic liver injury (Stancic et al., 2022). These effects are linked to the inhibition of the HMGB1–TLR4–NLRP3 signaling cascade and restoration of GPX4/SLC7A11 expression. AF exerts dual roles in ferroptosis and ICD by inhibiting TrxR1 and GPX4, especially in cancers with mutant p53 [e.g., NSCLC (Boullosa et al., 2021) and GBM (Wang M. et al., 2025)]. AF enhances HMGB1 and CRT release, boosts DC activation, and improves ICB efficacy. RSV, a plant-derived polyphenol, inhibits HMGB1 release by activating SIRT1 and miR-149, reducing oxidative stress and ferroptosis in diabetic retinopathy (Pen et al., 2025) and myocardial injury (Wang X. et al., 2022). In NSCLC-associated cancer-associated thrombosis (CAT), RSV reduces HMGB1-enriched EV release and platelet ferroptosis (Zhang et al., 2025).

In addition to classical ferroptosis inhibitors such as Fer-1 and Lip-1, several novel therapeutic strategies have emerged targeting the ferroptosis–HMGB1 axis across different disease models. (−)-Epicatechin, for instance, offers neuroprotection in intracerebral hemorrhage by activating Nrf2 and suppressing AP-1–mediated ferroptotic and inflammatory cascades, accompanied by reduced HMGB1 expression (Chang et al., 2014). Similarly, iridium (III) polypyridine complexes, as mitochondria-targeted photosensitizers, induce ferroptosis and ICD through GPX4 suppression and robust HMGB1/CRT release (Niu et al., 2025). Moreover, microneedle-delivered sulfasalazine–Mn nanoplatforms have shown promise in enhancing ferroptosis and adaptive immunity in postoperative tumor therapy, with potent HMGB1-mediated immune activation (Wang et al., 2024d).

Recent photodynamic therapy (PDT) approaches have also been explored to induce ferroptosis and ICD. 5-Aminolevulinic acid (ALA)-based PDT, for instance, induces LPO and modulates HMGB1 and ATP levels in esophageal cancer cells, although its effect on macrophage phenotype remains to be clarified (Čunderlíková et al., 2024). Notably, clinically approved PDT agents such as photosens and photodithazine have demonstrated strong ICD-inducing capacity through ferroptosis-associated HMGB1 release and DC maturation (Turubanova et al., 2019). Similarly, porphyrazine-based photosensitizers (e.g., pz I and pz III) effectively elicit immunogenic ferroptotic death and subsequent antitumor immunity via HMGB1 and ATP release (Turubanova et al., 2021). These studies highlight the potential of PDT-enhanced ferroptotic ICD as an adjunct to immunotherapy.

Additionally, recent evidence confirms that early ferroptotic cancer cells, characterized by high HMGB1 and ATP release, promote DC maturation and induce a vaccination-like effect in immunocompetent mice, demonstrating the time-dependent immunogenicity of ferroptosis (Efimova et al., 2020). To address the immunosuppressive effects caused by ferroptosis-induced HMGB1 release, dual-modality nanoplatforms have been developed. For instance, GA-loaded FeOOH–DMOS nanospindles induce GSH-dependent ferroptosis while concurrently inhibiting HMGB1 release, leading to enhanced ICB efficacy in tumor models (Sha et al., 2025).

### 4.2 Emerging therapeutic strategies

Nanomedicine platforms have greatly enhanced delivery specificity and synergistic modulation of ferroptosis and HMGB1. BQR@MLipo, a mitochondria-targeted liposomal platform, induces ferroptosis and HMGB1/mtDNA release in bladder cancer, activating cGAS–STING–IFNβ signaling and enhancing CD8^+^ T cell responses (Ding et al., 2023). T-LMD (cRGD-Magnetoliposomes) facilitates DOX and iron delivery to tumors, inducing ferroptosis and HMGB1 release, promoting DC activation and antitumor immunity (Shetake et al., 2024). Other physical delivery modalities such as microneedles (Wang Y. et al., 2024d) and histotripsy (Pepple et al., 2023) have been utilized for localized induction of ferroptosis and ICD in solid tumors.

Several natural or traditional Chinese medicine (TCM) formulations modulate HMGB1 release and ferroptosis via anti-inflammatory and antioxidative signaling. Da Cheng Qi Decoction (Chen et al., 2025) and Xiao-Ban-Xia Decoction (Liang et al., 2024) inhibit HMGB1 release by modulating NOX2/GPX4 or Nrf2 pathways in pancreatitis and chemotherapy-induced nausea. OY (Gao et al., 2024) attenuates ferroptosis in ulcerative colitis by suppressing HMGB1 and related inflammatory mediators (*HIF1A, IDO1, DUOX2*). Combination therapies, such as SGLT2 (Liu C. et al., 2024) and DPP-4 inhibitors (Nomura et al., 2024), also suppress platelet-derived HMGB1 release and alleviate ferroptosis-driven inflammation in diabetes.

Immunomodulatory strategies targeting HMGB1 include HMGB1-neutralizing antibodies (Davaanyam et al., 2024), which reduce ferroptosis and neuroinflammation by disrupting HMGB1–TLR4–hepcidin–iron overload signaling in stroke models. Epigenetic interventions, such as METTL3-mediated m^6^A modification of HMGB1 mRNA, alter ferroptotic sensitivity in osteoarthritis and malignancies (Bao et al., 2024). Additionally, chronotherapy, aligning drug administration with circadian regulators (e.g., BMAL1), has demonstrated therapeutic benefits in AML by modulating ferroptosis-associated immune dysfunction (Hong Zheng et al., 2024; Table 2).

**TABLE 2:** Directly or indirectly HMGB1-Targeting ferroptosis modulators in systemic diseases.

Modulator	Disease	Mechanisms and disease outcome	References
GLY	ALF	Inhibits HMGB1, activates Nrf2/HO-1/GPX4 axis, reduces liver injury	Wang et al. (2019)
	Neonatal HIBD	Inhibits HMGB1 and lipid ROS accumulation, restores GPX4, alleviates neuronal ferroptosis and inflammation	Zhu et al. (2022)
	Myocardial IRI	Inhibits HMGB1-TLR4 signaling, restores GPX4, improves cardiac function	Zhu et al. (2024)
GA	MTX-induced Hepatotoxicity	Inhibits HMGB1-mediated ferritinophagy, reduces ferroptosis, attenuates liver injury	Wang et al. (2024b)
	Ischemic Stroke	Inhibits HMGB1-ALPK1 interaction, suppresses pyroptosis and ferroptosis, reduces brain infarct size	Du et al. (2025)
	AKI-CKD Transition	Inhibits extracellular and intracellular HMGB1, improves tubular cell resilience; reduces long-term fibrosis and hypoxia, attenuates AKI-CKD progression	Zhao et al. (2023b)
Iridium *(III)* complexes	Melanoma, lung cancer	Elevates HMGB1, CRT, and HSP70 release, induces ferroptosis and apoptosis via mitochondrial damage, enhances anticancer efficacy	Gechang et al. (2024), Niu et al. (2025)
SA	Lower Limb IRI	Suppresses HMGB1 expression, reduce ferroptosis, alleviates muscle injury	Fengdan Wang et al. (2023)
Kaempferol	Skin Flap IRI	Activates SIRT1 to inhibit HMGB1/TLR4/NF-κB pathway, suppressing ferroptosis, improves flap viability and tissue repair	Wang A. et al. (2025)
Ethyl pyruvate	AKI-CKD	Inhibits HMGB1 to improve resilience of tubular cells, promoted renal recovery	Zhao et al. (2023b)
Esketamine	LPS-induced neurotoxicity	Inhibits ferroptosis, reduces and HMGB1, Attenuated neuronal injury	Wang J. et al. (2022)
*Gastrodia elata* polysaccharide	CIRI	Upregulates Nrf2/HO-1 pathway, enhances GPX4, SOD, GSH; reduces ROS, MDA, Fe^2+^, inhibits HMGB1, NLRP3 and proinflammatory cytokines, Reduced infarct size, improved neurological function	Zhang et al. (2024)
Microneedle patch	Breast cancer, colon cancer	Promotes HMGB1 release and induces ferroptosis, suppresses tumor recurrence	Wang et al. (2024d)
Jianpi-Tongluo Formula	OA	Inhibits NCOA4-mediated ferritinophagy, HMGB1 signaling, and ferroptosis, reduces cartilage degeneration, joint edema	Liu Y. et al. (2024)
T-LMD	TNBC	Induces ferroptosis leading to HMGB1 release, enhances antitumor immunity	Shetake et al. (2024)
Fer-1	Allergic Asthma	Inhibits ferroptosis and LPO; restores GPX4 and reduces HMGB1, IL-33, ALOX15, Alleviated AHR, inflammation, and airway remodeling	Li Y. et al. (2023)
	PH	Inhibits endothelial ferroptosis and inflammation by downregulating HMGB1-TLR4-NLRP3 pathway, reduces vascular remodeling	Xie et al. (2022)
	Cerebral IRI	Suppresses ferroptosis, reduces HMGB1 and NF-κB expression, reduces infarct size, neuronal injury	Chen Y. et al. (2023)
	Cardiomyopathy	Inhibits ferroptosis and HMGB1 expression, improves survival and cardiac function	Zhang et al. (2021)
	CKD	Inhibits ferroptosis, restores GPX4 and GSH/GSSG balance, reduces COX2, HMGB1, mproves tubular injury	Wang et al. (2023a)
	PAD	Inhibits LPO and HMGB1 release, reduces ferroptosis and inflammation in PAD.	Lillian Tran et al. (2023)
	Diabetic Liver Injury	Reduces oxidative stress and ferroptosis by restoring GPX4, SOD, HO-1, SLC7A11, and suppressing HMGB1 translocation, improves liver function	Stancic et al. (2022)
	SJS/TEN	Reduces ferroptosis, mitigates drug-induced epidermal damage and systemic inflammation	Lin et al. (2024)
Lip-1	Neutrophilic Asthma	Inhibits ferroptosis by restoring GPX4/SLC7A11, reduces HMGB1, suppresses pro-inflammatory cytokines, alleviates airway inflammation, mucus secretion, and histopathology in neutrophilic asthma	Bao et al. (2022)
	RA	Prevents ferroptosis of M2 macrophages, inhibits HMGB1-TLR4-STAT3 signaling and restores M1/M2 balance, reduces synovial inflammation and joint destruction in RA.	Feng et al. (2024)
Da Cheng Qi Decoction	SAP	Inhibits ferroptosis via NOX2 suppression and GPX4 upregulation, reduces ROS and HMGB1, alleviates pancreatic injury, improves SAP prognosis	Chen et al. (2025)
N6F11	PDAC	Induces ferroptosis and triggers HMGB1-mediated antitumor immunity, enhances antitumor immunity	Jingbo Li et al. (2023)
Celastrol	Hepatic Fibrosis	Inhibits PRDXs antioxidant activity, induces ferroptosis, attenuates liver fibrosis	Luo et al. (2022)
ML355	Lung IRI	Inhibits ferroptosis; blocks HMGB1 release, alleviates lung injury	Chongwu Li et al. (2024)
LxA4	Lung IRI	Activates FPR2 signaling, restores GPX4/Nrf2, reduces HMGB1, improves lung compliance	Sharma et al. (2022)
RvD1	AAA	Inhibits NOX2/p47phox via FPR2, reduces LPO and HMGB1 release, preserves aortic structure and immune homeostasis	Amanda et al. (2022)
AF	NSCLC	Inhibits TrxR1 and GPX4, induces ferroptosis, increases LPO, DNA damage, triggers ICD via DAMPs release, promotes DC maturation and NK-mediated cytotoxicity	Boullosa et al. (2021)
	GBM	Inhibits TrxR1, induces ferroptosis, suppresses tumor growth, prolongs survival	Wang M. et al. (2025)
BQR@MLipo	bladder cancer	Induces ferroptosis, promotes ICD, enhances CD8^+^ T cell infiltration and improves CBI antitumor efficacy	Ding et al. (2023)
RSV	NSCLC-related CAT	Promotes HMGB1-enriched EVs release, induces platelet ferroptosis, suppresses platelet activation, reduced CAT risk	Zhang et al., 2025
	Myocardial injury	Upregulates miR-149 to suppress HMGB1, inhibits ferroptosis in cardiomyocytes, improves cardiac function	Wang X. et al. (2022)
	Diabetic retinopathy	Activates SIRT1 to inhibit HMGB1 acetylation, reduces ROS and inflammation, restores GPX4/SLC7A11 expression, suppresses ferroptosis, improved retinal structure and function	Pen et al. (2025)
Fisetin	CKD	Inhibits tubular ferroptosis, reduces COX2 and HMGB1, attenuates tubular injury	Wang et al. (2023b)
OY	UC	Inhibits ferroptosis and inflammation, alleviates colon damage	Gao et al. (2024)
Xiao-Ban-Xia Decoction	CINV	Activates Nrf2 pathway, inhibits ferroptosis and HMGB1-mediated inflammation, attenuates gastrointestinal damage and suppresses CINV.	Liang et al. (2024)
(−)-Epicatechin	ICH	Activates Nrf2, reduces HMGB1 release, inhibits ferroptosis, protects against neurodegeneration	Chang et al. (2014)
Acteoside	HIRI	Inhibits HIF1α/p300–HMGB1 signaling, reduces ferroptosis and macrophage recruitment	Jia et al. (2025)
Isoliquiritigenin	AKI	Inhibits ferritinophagy, restores GPX4/SLC7A11 levels, protects against LPS-induced renal tubular injury	Tang et al. (2021)
ZINC006440089	ICH	Blocks HMGB1-mediated suppression of Nrf2/HO-1 signaling, prevents endothelial ferroptosis and BBB disruption, reduces hemorrhagic transformation	Liu J. et al. (2024)
Senkyunolide I	Solar dermatitis	Inhibits autophagy-dependent ferroptosis and HMGB1-TfR1 feedback loop to prevent ferroptosis cascade and inflammatory skin damage, protects against solar dermatitis	Wei et al., 2024
Shikonin	MM	Induces ferritinophagy and ferroptosis, leading to HMGB1 release, triggers ferroptosis and ICD.	Li et al. (2022)
DXZ	DOX-induced cardiotoxicity	Reduces DOX-induced ferroptosis and HMGB1 expression, improves cardiac function	Zhang et al. (2021)

### 4.3 Potential limitations and adverse effects

Targeting the ferroptosis–HMGB1 axis shows therapeutic promise but also has potential limitations and adverse effects. HMGB1’s dual roles mean its inhibition could cause immunosuppression and increased infection risk. Ferroptosis inhibitors might disrupt normal physiological processes and mitochondrial function. Off-target effects of therapeutic agents could lead to unintended side effects. The therapeutic window may be narrow, requiring precise dosing and timing.

## 5 Conclusion and future perspectives

The ferroptosis-HMGB1 axis has emerged as a critical mechanism linking redox imbalance, cell death, and immune regulation across numerous systemic diseases. HMGB1 acts as both a trigger and amplifier of ferroptosis, engaging receptors such as TLR4 and RAGE to propagate inflammation and tissue injury. This reciprocal relationship forms a vicious cycle that exacerbates disease progression in respiratory, digestive, nervous, circulatory, and other systems.

A growing repertoire of pharmacological modulators ranging from classical ferroptosis inhibitors (e.g., Fer-1, Lip-1) and HMGB1 antagonists (e.g., glycyrrhizin) to dual-function agents and advanced nanomedicine platforms has demonstrated promising efficacy in experimental models. Notably, recent advances in delivery systems such as mitochondria-targeted liposomes, microneedles, and hypoxia-sensitive nanocarriers have further enhanced the precision and efficacy of targeting this axis.

Future research should focus on refining the mechanistic understanding of ferroptosis-HMGB1 interactions, particularly in relation to subcellular dynamics, post-translational modifications, and tissue-specific regulatory networks. Biomarker-guided precision therapy, chronopharmacological approaches, and rational combination regimens (e.g., ferroptosis inducers with ICB) are promising avenues for advancing therapeutic efficacy. Importantly, longitudinal studies assessing safety, delivery optimization, and immune tolerance will be critical for clinical development.

In conclusion, the ferroptosis-HMGB1 axis represents a mechanistically rich and clinically actionable target for the treatment of diverse systemic diseases. Continued interdisciplinary efforts bridging redox biology, immunology, pharmacology, and nanotechnology will be essential for translating these conceptual advances into effective and durable therapies.
